# Effects of Self-Construction of Materials on the Ecological Awareness of Physical Education Primary School Students

**DOI:** 10.3390/ijerph192114176

**Published:** 2022-10-30

**Authors:** Paula Botella, Salvador Baena-Morales, Olalla García-Taibo, Alberto Ferriz-Valero

**Affiliations:** 1 Department of General and Specific Didactics, Faculty of Education, University of Alicante, 03690 Alicante, Spain; 2 EDUCAPHYS Research Group, Department of General and Specific Didactics, Faculty of Education, University of Alicante, 03690 Alicante, Spain; 3 Department of Physical Education and Sport, Pontifical University of Comillas, CESAG-Mallorca, 07013 Palma de Mallorca, Spain

**Keywords:** sustainable development, physical education, SDG, recycle, reuse, education for sustainable development

## Abstract

Physical Education (PE) has unique features to expand the students’ sustainability awareness. Being active in natural environments has been described as an opportunity to contribute on this matter. However, there is lack of related research. Therefore, the aim of this study was to evaluate the effect of a didactic proposal for PE based on the self-construction of materials on the ecological awareness of primary school students. A total of 111 students participated in this quasi-experimental study that lasted 4 weeks (eight sessions). The Mann-Whitney U test was applied for comparisons between groups, experimental and control, (SELF vs. CON) on two factors (PRE and POST). No significant differences were observed in any of them (*p* ≤ 0.05). On the other hand, to compare the data from the pre and post questionnaires within the same group (PRE vs. POST), the Wilcoxon signed-rank test was used. No significant differences in any of the groups were observed (*p* ≤ 0.05). Although SELF improved on some scores such as reusing things after picking up litter from yards, the differences were not significant. This could be explained by the brevity of the intervention. These results could contribute to increasing research on the integration of sustainability in PE.

## 1. Introduction

### 1.1. Sustainable Development in Today’s World

“Climate change is not a problem of the future: it is already producing changes that will be irreversible for centuries or millennia” [1] (p. 21). These words were uttered at the last meeting of the United Nations panel of experts on climate change. However, this issue is not new. For years, geologists have been claiming that human activity is capable of causing major changes in the earth’s surface and in the composition of the atmosphere [2]. In fact, this is the main cause of most contemporary environmental changes. Although this period has been short, speaking in geological time scales, it has been powerful enough to consider the present within a new geological era, the Anthropocene, leaving behind the Holocene Era [3]. As Zalasiewicz et al. [4] pointed out, “human reign has been geologically brief, but so was the meteorite impact that probably ended the world of the Cretaceous Era. In this case, we are collectively that meteorite” (p. 181). “Our world is in danger” [5] (p. 4). Temperatures continue to rise, devastating weather events, sea levels, acidification and water pollution are increasing, and more and more species are becoming extinct. In addition to all these environmental problems, climate change also has negative effects on human health, energy production and access to resources. These are consequences that are mainly reflected in the world’s most vulnerable and poorest people, thus increasing inequality, social unrest and conflict [5].

Looking at the Earth’s environmental situation, our greatest challenge is to respond to what the human actions of the last century have led to. Such a response is called sustainable development. Sustainable development is understood as “development that meets the needs of the present without compromising the ability of future generations to meet their own needs” [6] (p. 41). It seeks the progress of humankind towards the common good in a way that is fair and responsible towards future generations and nature [7]. However, sustainability should not only be seen from an environmental perspective, because, as mentioned above, the problems do not stop there, but also extend to a social and economic dimension. [8]. Fortunately, there seems to be a globally positive trend towards a more sustainable society. This is observed in the Sustainable Development Goals (SDGs), adopted as a global commitment in the climate negotiations within the United Nations [9]. These define 17 goals divided into 169 targets to be implemented in the new post-2015 development agenda. They address poverty eradication, as well as responsible economic development; environmental limits, climate change and the use of natural resources to protect the environment, and action on a range of social issues: inequality, education, health, job opportunities, protection, etc. These are goals “of the people, by the people and for the people” [10].

### 1.2. Education for Sustainable Development and Physical Education

It is time for everyone to put the SDGs into action: governments, the private sector, civil society and every human being has to do their part [11]. Campos-López & Contreras [12] explained that “Beyond the harmful effects of economic imbalance, the real problem lies in the separation of the self from the environment or, in other words, in the lack of awareness of the impact of our actions on the environment” (p. 58). The main way to raise awareness among the younger generation is through education. This calls for Education for Sustainable Development (ESD) that develops students’ competences to reflect on their own actions and make responsible decisions taking into account their current and future environmental, social, and economic consequences [11]. As can be seen, the United Nations established SDG 4 “quality education” as a fundamental axis to guarantee the achievement of the SDGs. The UN Convention on the Rights of the Child declared that education should inculcate respect for the environment [13]. In view of this, the importance of schools developing ESD during all educational stages is confirmed once again, in order to pursue a society conformed by people educated in sustainability who are committed to sustainable solutions [14,15]. However, in order to achieve this level of awareness and commitment, an education that uses active and participatory methodologies that activate the affective aspects of students is necessary [15,16]. There are numerous experiences that demonstrate the possibility of promoting sustainable development in the educational context, specifically in primary education. Moreover, these have been carried out in different areas, since sustainability and care for the environment are cross-cutting elements that should be approached from the methodological principles of the different subjects [17,18,19]. The 2030 Agenda also recognizes sport as an “important enabler of sustainable development”. In fact, as the Ibero-American Sports Council [20] pointed out, “sport has historically played a very important role in all societies” (p. 11). Moreover, in 1978, UNESCO described PE as an indispensable element in the global development of the child [21]. As such, the PE provides opportunities for young people to acquire a range of values, skills and abilities that are relevant to their lives, and to new forms of global citizenship that contribute to sustainable development. In addition, the Charter for Quality Physical Education states that it should provide a variety of contexts and environments that require resourcefulness and creativity on the part of students, develop transferable skills to support achievement beyond the curriculum, and foster environmental responsibility [21]. Although the role of PE is not specifically mentioned in the 2030 Agenda, Baena-Morales et al. [22] explained which SDGs and targets can be developed through PE and related them to the different models based on PE practices. Of the 169 SDG targets, they indicated that 24 could be developed on in this subject. Within the different aspects of sustainable development to which PE can develop, this study could contribute to targets 12.2 (efficient use of natural resources), 12.5 (reduce, recycle and reuse), 12.8 (knowledge for sustainable development) and 13.3 (climate change education and awareness) of SDGs 12 (responsible production and consumption) and SDGs 13 (climate action) [10].

With the aim of developing these goals through a didactic proposal for primary education, the pedagogical model based on the self-construction of materials will be used [23]. This means collecting, recycling, handling, and transforming raw and homemade materials to develop teaching materials for class [24]. PE teachers perceive that the use of self-made materials contribute on a cognitive, physical-motor, affective and values level of the students [25]. Moreover, creating new resources for the PE class by reusing materials promotes the development of an ecological and responsible awareness, as well as personal values and attitudes that benefit the achievement of these goals [26]. However, this is not the only reason why PE teachers use this model in their classes. This model appears as a solution to the limited funds and lack of sports equipment in many schools [27], it enables the creation of adaptable materials [28], it increases motivation, enjoyment and student interest [28,29,30], and the approach of this model allows the easy hybridization with other methodologies [23]. In addition, previous research on the self-construction of materials has shown how this model, in the school context, enables a greater availability of material that helps students to increase their motor experiences in PE and increase activity practice [31]. It has been pointed out that this procedure produces greater interest, enjoyment and enthusiasm from students, who can be assumed to be more physically active in PE classes and even outside school [32,33]. Even in the case of students having difficulties in practicing PE content in their free time due to economic barriers and lack of materials [34], self-construction of material could be a solution [25]. Furthermore, these ideas are in line with the Global Plan of Action on Physical Activity 2018–2030 proposed by the World Health Organization (WHO) [35], pointing out the importance of promoting the practice of physical activity with respect for the environment and sustainable development of the planet.

Finally, it should be noted that building materials for sports practice in PE is not the content to be developed as the central goal, but will be a tool to educate in attitudes and values related to the reuse of materials, in order to develop competences in students that lead them towards a more sustainable life. “We must understand our subject not as an end in itself but as a means to help create a more prosperous and sustainable world” [26] (p. 13). Furthermore, according to the stipulations of Royal Decree 157/2022, of March 1, establishing the organization and minimum teachings of Primary Education in Spain [27], PE is an ideal subject to work on the self-construction of material in its sessions. Specifically it points it out as “Valuing different natural and urban environments as contexts of motor practice, interacting with them and understanding the importance of their conservation from a sustainable approach” (p-54).

## 2. Materials and Methods

### 2.1. Research Design and Objectives

A quasi-experimental study was carried out for this research. The intervention with Primary Education students in the PE area was based on the construction of sports materials using waste and reusable elements, such as plastic bottles, bags or newspaper. The main objective of the work was (1) to evaluate the effect of an intervention in the PE area based on the self-construction of materials on ecological awareness. Specifically, it was to evaluate the pro-environmental attitude related to recycling, reuse, pollution and preservation of the environment. Secondarily, the aim was also (2) to analyze how primary school pupils value the experience of building their own material for the PE area and its use in sports initiation sessions.

### 2.2. Participants

The educational intervention was carried out in the 6th year of primary school in two different schools. Therefore, a sample of 124 subjects was estimated. Finally, due to some absences in certain sessions, there were 13 dropouts, so the final sample was 111 participants. In terms of gender, 47.75% of the total sample were female, 51.35% male and 0.90% preferred not to say. The mean age of the students was 11.20 years, with a standard deviation of 0.40. Before starting the intervention, all participants were informed of the objectives of the study and that they could quit at any time. Since the subjects were minors, their mothers, fathers or legal tutors signed an informed consent form authorizing their participation and the transfer of their data for scientific use. The study design respected the ethical aspects presented in the Declaration of Helsinki. This research was approved by the ethics committee of the University of Alicante with code UA-2022-03-17.

### 2.3. Procedure

**Figure 1:**
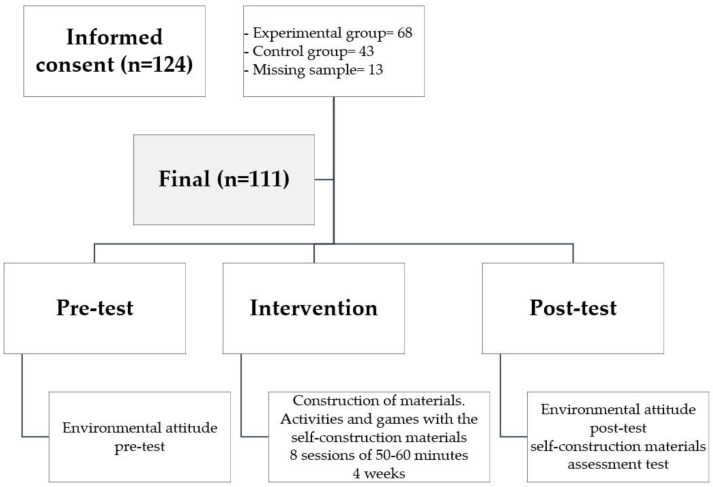
Summary of the procedure.

A pragmatic quasi-experimental design was conducted, emphasizing its ecological validity by placing the research in a real context. The study was based on implementing a didactic intervention in which the methodology used was the self-construction of materials, and on assessing the students’ pro-environmental attitude before and after the intervention by filling out a questionnaire previously validated. In addition, after the development of the proposal, a questionnaire was completed to evaluate the experience with the self-constructed materials. The proposal consisted of eight sessions per group, two per week, with a duration of 60 min in one of the schools and 50 min in the other, coinciding with the duration of a didactic unit programmed by the teachers. The duration of these sessions is in agreement with that presented in previous literature [24,25]. In order to assess whether the intervention really had an effect, the sample was divided into two groups, an experimental group (SELF), which worked with the proposal based on the self-construction of materials, and a control group (CON), which followed the normal PE routine based on the practice style and with non-self-constructed materials. Specifically, performed activities with technical, tactical and physical components related to a collective sport. The division of groups was not randomized, as the establishment of groups per grade in the school was respected. In the school that had 3 classes per grade, there were two experimental and one control group; and in the school which had 2 classes per grade, there was one experimental and one control group. Therefore, of the total research sample, 68 were part of SELF and 43 were part of CON. With regard to gender, in both SELF and CON there was a balance in both groups. In SELF, 47.06% were female, 51.47% male and 1.47% preferred not to say. In CON, 48.84% were female and 51.16% male. A summary of the procedure is shown in Figure 1.

**Figure 2:**
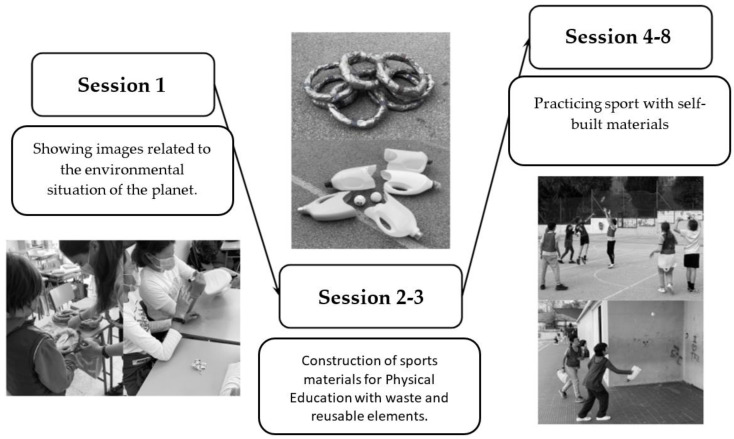
Infographic summary of the intervention.

The intervention based on self-constructed materials considered previous literature for its design [36,37]. An initial session, separate from the intervention, was devoted to carrying out the initial assessment (filling in the pre-test that measures the pro-environmental attitude) and to providing information to the students about what we were going to do and the reasons why. For this purpose, we undertook an activity in which a guided group discussion was held using pictures. These photographs were related to the severe situation of the planet, consumption, pollution, and the human ecological footprint, so that the students could share what they knew about the impact to people on Earth. Finally, the issues discussed were related to the contents of the following session, which involved self-constructing materials with reusable materials to be used in the PE sessions. During the second and third session, didactic materials for the PE subject were constructed with reusable elements. Previously, given sufficient time, the students had been asked to collect these elements at home. The materials that were constructed were flying hoops made from newspaper, plastic bags and tape, and baskets for catching balls made from detergent containers. In the following five sessions, the materials made were used to conduct different activities and games related to ultimate sports and a form of Pelota Vasca in which baskets are needed. These sessions also aimed to develop the generic skills of throwing and catching. The last session (nine session) consisted of completing the post-test by measuring pro-environmental attitudes. Another questionnaire was also filled in to assess the use of self-constructed materials. Finally, Figure 2 presents a more detailed summary of the intervention performed. In relation to the quality and duration of the self-constructed materials during the process, it was observed how 100% of the materials were able to resist the whole intervention. In fact, six months after the intervention, the teachers responsible for the centre were asked about the state of the materials, confirming that they were still in a suitable state for use.

### 2.4. Instruments

A questionnaire previously validated was used to assess whether the intervention has affected the pupils’ environmental awareness. This is the Scale for Measuring Children’s Environmental Attitudes (EMAPI) [38], which has a high reliability since its Cronbach’s Alpha coefficient is α = 0.626. It consists of 10 items evaluated by a 4-point Likert-type scale, which registers the degree of pro-environmental attitude (1 indicating the lowest attitude and 4 the highest attitude). The items measure two different dimensions or attitudes:

Environmentalism (items 1, 7, 8, 9 and 10) relates to conservation, recycling and pollution issues. For example, item 9 states that “Some children do not separate rubbish but other children separate rubbish and recycle it”.

It should be noted that of the two dimensions measured by the scale, the intervention aimed to improve environmentalism, because the didactic proposal deals with the reuse of different materials. Therefore, the other issue, animal rights, was not addressed. The format of the questionnaire is a “version” of the typical Likert-type scale adapted to children. Thus, instead of circling a number, they had to identify themselves with one of the descriptions of the two types of children that appeared, and then fill in a large square if the statement is always true or a small square if the statement is not always true. It should be added that some words have been adapted because of the differences between the Spanish vocabulary used in Venezuela, where the scale comes from, and that used in Spain (Annex 3). The questionnaire was completed by both groups, SELF and CON, both before (pre-test) and after (post-test) performing the didactic proposal.

For the assessment of the experience, the “Assessment of the use of self-built materials in PE” questionnaire [39] was used. This consists of 15 items related to the possible difficulties encountered, the effort made, the level of enjoyment, the degree of motivation or the usefulness of the material to improve their skills, among others. The items are evaluated on a 5-point Likert-type scale (1 indicating “no, not at all”, 2 “yes, somewhat”, 3 “yes, regularly”, 4 “yes, quite a lot” and 5 “yes, a lot”). It should be noted that the last item is scored differently, as the students have to decide whether they would have had more fun playing with the conventional material or with a constructed material, so there are only two response options (1: “I would have had much more fun with a purchased material”, and 5: “I had much more fun with the constructed material”). Since in this intervention two types of materials have been constructed, it is worth mentioning that some items have been divided into two sections, one for each type of material (Annex 4). This questionnaire has only been filled in by the experimental group (SELF), as it was the one who carried out the intervention with these materials.

### 2.5. Data Analysis

The data obtained through the questionnaires measuring the pro-environmental attitude were entered and analyzed using the statistical programmes Statistics Product and Service Solutions (IBM^®^ SPSS^®^ Statistics Version 24.0.0.0) (International Business Machines Corp., Madrid, Spain) and Microsoft Excel^®^ ( 2016 version) (Microsoft Corp., Redmond, WD, USA). Considering that the SELF sample was larger than 50 subjects, the Kolmogorov-Smirnov test was used to assess the normality of the continuous variables of the set, obtaining values of *p* < 0.05, in all of them. On the other hand, for the CON sample, with less than 50 subjects, the Shapiro Wilk test was considered, whose values were also *p* < 0.05. Since normality tests indicated that the data were non-parametric, they were further subjected to chi-square analysis and univariate statistical analysis for non-parametric samples. The Mann-Whitney U test was used to assess differences between groups (SELF vs. CON) on two occasions: pre- and post-intervention; and the Wilcoxon signed-rank test was used to assess intragroup differences (PRE vs. POST). The significance level was set at *p* < 0.05 in all cases. According to Faul et al. [40], the statistical power of the sample size was calculated using the free software G*Power (to see. 3.1.9.6, University of Dusseldorf, Germany). The sample size was 68 participants in the experimental group and 43 participants in the control group, with an estimated medium effect size (0.5), and a significance of 95%, resulted in a power of 0.99.

## 3. Results

### 3.1. Descriptive Analysis of the Results of the EMAPI Questionnaire

As mentioned above, the questionnaire used as an instrument to measure pro-environmental attitudes in children (EMAPI) was completed twice by the subjects, once before the educational intervention (pre-test) and once after the intervention (post-test). Table 1 records the means and standard deviations for each item. In relation to environmentalism, SELF registers improvements in items 1 (recycle things that can no longer be used), 7 (reuse things after use) and 8 (pick up rubbish from the yard). CON also improves in items 1 and 8 but to a lesser extent. In items 9 (separating rubbish) and 10 (living in the countryside without pollution), SELF worsens with a difference of 0.04 and 0.01, respectively.

### 3.2. Comparative Analysis of the Results of the EMAPI Questionnaire

As mentioned above, to assess the normality of the continuous variables in the sets, the data were analysed using the Kolmogorov- Smirnov and Shapiro Wilk tests. In both tests, values of *p* < 0.05 were obtained, so it was concluded that the sample was non-parametric. Therefore, the non-parametric Mann-Whitney U test and the Wilcoxon signed-rank test were used to measure the effect of the intervention. With the Mann-Whitney U test, comparisons were made between the groups (SELF vs. CON) on the two factors (pre and post) measured by the questionnaire. However, as can be seen in Table 2, no significant differences were observed in any of them, since a value of *p* < 0.05 was obtained.

Next, in order to compare the data obtained from the pre and post questionnaires within the same group (PRE vs. POST), the Wilcoxon signed-rank test was used. However, there were no significant differences in any of the groups, since values of *p* > 0.05 were obtained, as shown in Table 3.

### 3.3. Descriptive Analysis of the Results of the Questionnaire on the Use of Self-Built Materials

In relation to the questionnaire “Assessment of the use of self-built materials in PE”, Table 4 shows the means and standard deviations of each of the items related to the construction of materials for the total sample.

**Figure 3:**
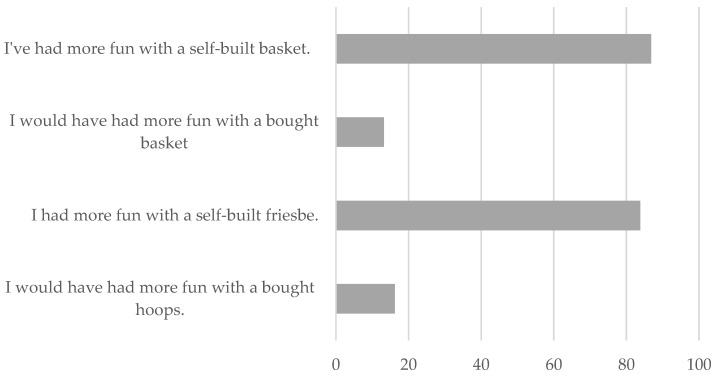
Bar chart to compare the level of enjoyment as a function of the material built or purchased according to the percentage of subject. Note. These percentages correspond to the answers to items 15.A (for the hoops) and 15.B (for the baskets).

The highest scores, in both materials, were obtained in questions 1, “Did you find it easy to find the materials to build the hoop or basket?”, 3, “Did you find the lessons you did with these materials fun?”, 5, “Did you find it useful to build the materials for the PE subject?”, and 7, “Did you like this experience of building your material for this subject?”. The lowest values were observed in items 2, “Did you find it hard to build the equipment?”, 8, “Would you use the hoop or basket to play at break time?”, and 9. Finally, Figure 3 refers to item 15. It shows the percentage of subjects who think that it is more fun to play with constructed materials or with purchased materials. It was found that, for both materials, the vast majority had more fun with self-built materials.

## 4. Discussion

The main objective of this research was to evaluate the effect of a didactic proposal based on the self-construction of materials on the pro-environmental attitude of primary school students in the area of PE. A lack of scientific literature on how PE can contribute to the development of sustainability has been detected, specifically with regard to its environmental dimension. However, some previous studies suggested that the characteristics of this area could improve it [22,23]. The data obtained did not indicate significant changes between students’ pro-environmental attitudes before and after the intervention. The results neither showed significant differences between the attitude of SELF and CON. This could be explained by the brevity of the intervention, as there were only eight sessions. Another reason could be that the students did not understand the proposal and its significance. Although part of the first session was dedicated to this target, perhaps this information could have been repeated in the rest of the sessions or even adding other information that could have had a greater impact on the subjects. Regardless of not having improved the environmental awareness of the students, since they used the self-made material and did not use new material, a contribution has been made to SDG 12 and target 12.5: “Reduce waste generation through prevention, reduction, reuse and recycling activities”. A similar study that evaluated the pro-environmental attitude of students aged 8–13 years after the implementation of a three-month environmental education workshop concluded that students showed a lower pro-ambiental attitude because they “analysed more and were more critical before giving their answer”, so that their answers were closer to reality [41]. It is worth noting that, in this research, although attitude did not improve, environmental behaviour did, perhaps due to the practicality of the workshop. Attitude is not the only element that can herald actions; aspects such as culture or knowledge also play a role [42]. As already mentioned, this study did not affirm that the didactic proposal has changed the pro-environmental attitude of the students. However, this information can be used to develop and test other types of educational interventions from considering and improving aspects not covered in this study. It was previously mentioned that one reason that could explain the absence of significant differences could be the brevity of the intervention. Changing attitudes and raising awareness may require a much longer process and involve more areas of education. In fact, Caduto [43] explains that an attitude is a relatively long-lasting organization of beliefs about an object or situation that predisposes a person to respond in a certain way. Furthermore, Benegas & Marcén [36] pointed out that the complex sets of attitudes of each individual make up his or her own unique scale of values; this, in turn, guides and determines his or her behaviour and way of life. Finally, they add that “the development of values is a social process and is progressively forged in people” (p. 13). This would require a more extended, constant and coherent work in all areas so that the pupil can really establish connections between what happens at school and what he/she can change in his/her daily life. In this case, it was not possible to extend the intervention further because of the lack of time available in the schools where it was carried out.

Furthermore, the lack of interest of most of the subjects in filling in the questionnaires could have had an impact on the results of the questionnaires, as they answered them quickly and without stopping to reflect. The lack of motivation in this situation may be related to the fact that they have to stay in the classroom for most of the day, so when it is time for PE they expect to go out to the playground. Moreover, the vast majority of pupils only associate PE with sport, which makes them even more disappointed, as they do not understand why they have to sit in a chair filling in a paper instead of going out and moving around. Future studies could consider implementing interdisciplinary proposals of longer duration that seek to promote environmental awareness among pupils. The fact that the questionnaires were completed in the playground or outdoors, or at any time of the day different from the PE sessions should also be taken into account in order to avoid prejudicing the mood of the subjects when they are filling in it. The second aim of the study was to analyze how primary school pupils rate their experience with the pedagogical model of self-construction of materials. Previous studies have shown how lower secondary school students enjoy building their own materials and using them in PE sports activities [39]. The results obtained in this research showed that the experience of building their material was quite easy, did not involve a great effort, and was enjoyable for use in sports practice. In addition, the vast majority of the sample felt that they had enjoyed using the constructed material more than they would have enjoyed the conventional material, which could be explained by the possible attachment that is generated towards the material after self-construction. However, in general, they stated that they would not use the constructed material at playtime or outside school. This could be because they were not given the opportunity to take the material to other spaces, or because they are currently engaged in other leisure activities that they find more rewarding. As for the other items, these were not highlighted at first glance. Comparing these data with others from a similar study carried out by Méndez-Giménez et al. [39] with secondary school students, it can be seen that in our study the score registered for item 4 “Has the material allowed you to improve your passing and receiving skills?” was much lower. This could be explained by the brevity of the intervention. This conditioning of time and, therefore, the duration of the proposal, is the main limitation detected.

After the study had been completed, it can be pointed out that the sixth year primary school pupils liked and enjoyed constructing their own material and using it in the practical sessions of the PE subject. This information can be very useful, since as many studies explain, this pedagogical model can be a great source of economical and adaptable resources [27,37]. Considering that the intervention implemented in the present study did not achieve the expected objective, the following are some potential limitations of this work. The first is related to the absence of previous studies on the self-build materials models which limited the orientations of the method to be followed on this study. So that, the data collection conducted did not include certain relevant aspects related to ecological awareness, knowledge and behavior of children in their daily lives. Therefore, future studies could include new questionnaires, qualitative tools and/or surveys. It could also be interesting to create and validate new questionnaires related to the topic of study. On the other hand, given the lack of previous literature on this topic, the present work could be interesting and useful, and therefore represents a line of research to be continued. Another limitation was the short duration of the intervention, eight sessions, since it was integrated into a didactic unit. One possibility for the future could be to develop an interdepartmental intervention with other subjects in which ecological awareness could be worked, in order to increase the training. Moreover, with the intention of not reducing movement time in the physical education class, the completion of the questionnaires could be integrated into homework after a correct explanation and clarification of the instrument. Finally, it should be remembered that another limitation of the study is its quasi-experimental nature, so a suggestion for the future is that it should be replicated by randomizing the assignment of the groups. Therefore, the self-construction of materials should be used more by teachers from the area. Although we do not know the lifespan of the material produced by the students, this material is still used in the PE sessions of the schools participating in the research. For future research, it could be interesting to measure the durability and lifetime of the recycled material. Also, the duration of the intervention should be taken into account, and a greater number of sessions should be devoted to the use and familiarization with the material. It would also be useful to study how the use of materials constructed for the subject area could be encouraged in other contexts different that PE. Finally, the hybridization of this model with others, such as cooperative learning [37] or service-learning [29], could be developed.

## 5. Conclusions

In response to the demands of the sustainability of the planet, a teaching proposal based on the pedagogical model of self-construction of materials, within the area of PE, could contribute to progress with students in SDGs 12 and 13, specifically with regard to goals 12.2, 12.5, 12.8 and 13.3. After the implementation of this proposal, no clear results have been obtained to confirm significant changes in the pro-environmental attitude of the subjects. However, the information provided can contribute to the development of other types of educational interventions by transforming and improving certain aspects. On the other hand, it can be affirmed that pupils, in the sixth year of primary school, liked and enjoyed the model of the self-construction of materials. This result should be considered by PE teachers in order to take advantage of a very successful resource for the area. In future research, it would be interesting to perform more extensive educational proposals covering more areas in a coordinated way, since changing attitudes and awareness in children requires time and coherence. It has been known for years that the place we live in is in danger, and only people can act to stop its destruction. If we want to respond to the message of help that our planet is asking for, now is the time to use the powerful tool we own as teachers.

## Figures and Tables

**Table 1:** Descriptive analysis for the EMAPI questionnaire in the pre and post phases for the control and experimental groups expressed as mean and standard deviation.

	Control		Experimental	
Items	Pre	Post	Pre	Post
**Factor 1. Environmentalism**				
Item 1. Recycle things that can no longer be used.	3.12 (0.70)	3.14 (0.74)	3.16 (0.78)	3.32 (0.84)
Item 7. Reuse things after using them.	3.14 (0.64)	3.09 (0.81)	3.12 (0.95)	3.22 (0.93)
Item 8. Pick up garbage from yards.	2.63 (0.87)	2.74 (0.85)	2.51 (0.91)	2.72 (0.81)
Item 9. Separate garbage	3.35 (0.95)	3.26 (1.00)	3.47 (0.87)	3.43 (0.94)
Item 10. Living in the countryside without pollution.	2.74 (1.07)	2.74 (1.11)	2.66 (1.07)	2.65 (1.09)

**Table 2:** Mann-Whitney U test results.

Environmentalism		
	Pre	Post
Z	−0.485	−1.106
Asymptotic sig.	0.628	0.269

**Table 3:** Wilcoxon signed-rank test results.

	CON					SELF				
	Positive	Negative	Ties	Z	Sig.	Positive	Negative	Ties	Z	Sig.
**Amb_postAmb_pre ^1^**	16	14	13	−0.052	0.958	34	21	13	−1.723	0.085

^1^ Differences in Factor 1: Environmentalism between the data collected in the post-test and in the pre-test.

**Table 4:** Descriptive analysis for the questionnaire “Assessment of the use of self-constructed materials in Physical Education”.

ITEMS	M	SD
1. A. Was it easy for you to find the materials to build the hoop?	3.46	1.33
1. B. Did you find it easy to find the materials to build the basket?	3.24	1.46
2. Did you find it difficult to build the material?	2.29	1.31
3. Did you find the lessons you did with these materials fun?	3.85	1.10
4. A. Did the hoops you built allow you to improve your passing and receiving skills?	3.10	1.20
4. B. Did the baskets you built allow you to improve your passing and catching skills?	3.34	1.22
5. Did you find it useful to build the materials for the Physical Education course?	3.40	1.33
6. Did the materials you built allow you to interact with your classmates?	2.96	1.33
7. Did you like the experience of building your own materials for this subject?	3.59	1.21
8. A. Would you use the hoop to play during recess?	2.31	1.36
8. B. Would you use the basket to play at recess?	2.37	1.41
9. A. Would you use the hoop to play outside of school?	2.34	1.41
9. B. Would you use the basket to play outside of school?	2.37	1.42
10. Has building the equipment encouraged you to practice and learn?	3.22	1.31
11. Do you think that building the equipment is related to the content of other subjects?	3.15	1.43
12. A. Would you like to continue to practice Ultimate with the constructed material?	2.82	1.26
12. B. Would you like to continue practicing *Pelota Vasca* with the constructed material?	3.00	1.32
13. Has this experience developed your creativity or imagination?	2.90	1.31
14. Do you think that using your built material has increased your desire to play?	2.78	1.21

## Data Availability

Not applicable.
